# Large Genomic Region Free of GWAS-Based Common Variants Contains Fertility-Related Genes

**DOI:** 10.1371/journal.pone.0061917

**Published:** 2013-04-17

**Authors:** Rong Qiu, Chao Chen, Hong Jiang, Libing Shen, Min Wu, Chunyu Liu

**Affiliations:** 1 School of Information Science and Engineering, Central South University, Changsha, China; 2 Hunan Engineering Laboratory for Advanced Control and Intelligent Automation, Changsha, China; 3 Department of Psychiatry, University of Illinois at Chicago, Chicago, United States of America; 4 Institute of Human Genetics, University of Illinois at Chicago, Chicago, United States of America; 5 Department of Neurology, Xiangya Hospital, Central South University, Changsha, China; 6 School of Life Science, Fudan University, Shanghai, China; 7 State Key Laboratory of Medical Genetics of China, Central South University, Changsha, China; University of Hong Kong, China

## Abstract

DNA variants, such as single nucleotide polymorphisms (SNPs) and copy number variants (CNVs), are unevenly distributed across the human genome. Currently, dbSNP contains more than 6 million human SNPs, and whole-genome genotyping arrays can assay more than 4 million of them simultaneously. In our study, we first questioned whether published genome-wide association studies (GWASs) assays cover all regions well in the genome. Using dbSNP build 135 data, we identified 50 genomic regions longer than 100 Kb that do not contain any common SNPs, i.e., those with minor allele frequency (MAF)≥1%. Secondly, because conserved regions are generally of functional importance, we tested genes in those large genomic regions without common SNPs. We found 97 genes and were enriched for reproduction function. In addition, we further filtered out regions with CNVs listed in the Database of Genomic Variants (DGV), segmental duplications from Human Genome Project and common variants identified by personal genome sequencing (UCSC). No region survived after those filtering. Our analysis suggests that, while there may not be many large genomic regions free of common variants, there are still some “holes” in the current human genomic map for common SNPs. Because GWAS only focused on common SNPs, interpretation of GWAS results should take this limitation into account. Particularly, two recent GWAS of fertility may be incomplete due to the map deficit. Additional SNP discovery efforts should pay close attention to these regions.

## Introduction

The human genome contains millions of common SNPs, which are being deposited into public databases. These data have been used to design genome-wide association studies (GWASs) [1], [2], [3]. Common SNPs are better powered in association tests [4]. However, genomic regions not covered by common variants are neglected. Those neglected regions may contain variants with low frequencies, and should be paid more attention to because rare variants are even more likely to be functional than common ones [5].

In our study, we were interested in two questions: 1) whether the human genome is sufficiently covered by common SNPs and is sufficiently captured by common SNPs of standard GWAS platforms, and 2) whether any genes were included in those regions and their enriched biological functions.

To answer these two questions, we started with searching regions without common SNPs, called common SNP-free regions (CSFRs), regions free of both common SNPs and CNVs, called common variant-free regions (CVFRs). Next, we explored the functional enrichment of genes identified in CSFRs and CVFRs. With available personal genome sequencing data, whether these CSFRs and CVFRs contain common and rare variants were also examined.

## Methods

### Identification of CSFRs and CVFRs

Common SNPs (MAF≥1%) in dbSNP build 135, Genome Assembly Gaps and Genome Database refGene data were downloaded from the UCSC Genome Browser (http://genome.ucsc.edu/) (Table 1). The CNV data were downloaded from the DGV (Table 1). Using the common SNP table, we calculated distances between adjacent common SNPs and subtracted regions containing the genome assembly gaps. If the remaining SNP intervals were longer than 100 kb, those intervals were defined as CSFRs. The CSFRs were further searched for CNVs. If after subtracting regions containing CNVs, the intervals were still longer than 100 kb, those intervals were defined as CVFRs. The reason we used 100 kb as bin to detect SNP free region is the SNP Linkage disequilibrium distance: several groups reported blocks of up to 100 kb in length exhibiting very strong linkage disequilibrium [6], [7].

**Table 1:** Data Sources Used in This Study.

Data	URL	Version	Modified date	Data description and summary statistics
Common SNP Data in HapMap	http://hgdownload.cse.ucsc.edu/goldenPath/hg19/database	Human Genome assembly hg19.	18-Dec-2011	snp135Common.txt.gz Total SNPs: 11,488,259 in chr1-chrY.
Genome Assembly Gaps data	http://hgdownload.cse.ucsc.edu/goldenPath/hg19/database	Human Genome assembly hg19.	27-Apr-2009	gap.txt.gz Total gaps, 357 in chr1-chrY.
Genomes Unzipped data	http://www.genomesunzipped.org/download/	Based on human genome hg18, upgraded to hg19	10-Oct-2010	Total of 1923 SNPs in the chrY.9 sample, 546 common SNPs with maf>1%.With data for 9 personal genome sequences.
personal genome variation data	http://hgdownload.cse.ucsc.edu/goldenPath/hg19/database/	Based on Human Genome assembly hg19.	21-Feb-2010	Total of 9 personal genomes: pgNA12878.txt.gzpgNA12891.txt.gzpgNA12892.txt.gzpgNA19240.txt.gzpgSjk.txt.gzpgVenter.txt.gzpgWatson.txt.gzpgYh1.txt.gzpgYoruban3.txt.gz
DGV data	http://hgdownload.cse.ucsc.edu/goldenPath/hg19/database/	Human Genome assembly hg19.	07-Mar-2011	dgv.txt.gz Total 101605 in chr1-chrY.
segmental duplication data	http://eichlerlab.gs.washington.edu/database.html	Human Genome assembly hg19.	27-Jun-2011	inter pairs is 22980; intra pairs is 8763
Genes	http://hgdownload.cse.ucsc.edu/goldenPath/hg19/database/refGene.txt.gz	Human Genome assembly hg19.	21-May-2012	Total number of genes is 42,742; after eliminating other chromosome, 30,332 genes in chr1-chrY remain.

To verify our result for its impacts on GWAS, we first determined whether the CSFRs are truly missed by Affymetrix Genome-Wide Human SNP Arrays. Next, we asked whether these regions included rare variations or were devoid of genetic variation. We analyzed common SNP data obtained from Genomes Unzipped (genomesunzipped.org) and Personal Genome Variation tracks from the UCSC Genome Browser. These two datasets are collections of variants that have been identified in the sequencing of personal genomes (Table 1).

### Identification of genes in CSFRs and CVFRs

Gene annotation data from the Human Genome assembly hg19 UCSC refGene was used to map coding genes in the CSFRs and CVFRs (Table 2). Genes were included if their transcription regions overlapped with the CSFRs/CVFRs by at least one base pair. When a gene had multiple splicing forms, we chose the longest splicing form to define the gene region.

**Table 2:** List of 50 common SNP-free regions containing 97 genes.

Chr	CSFR_start	CSFR_end	CSFR_size	Gene_name	Isochore_type
chr1	145883118	145989503	106385	GPR89C, PDZK1P1	Isochore_border
chr2	110524226	110704031	179805	RGPD5, RGPD6, LIMS3, LIMS3-LOC440895, LIMS3 L	Isochore
chr2	111191098	111347035	155937	LIMS3-LOC440895, LIMS3, LIMS3L, RGPD6, RGPD5	Isochore_border
chr7	74765724	74866460	100736	GATSL2	Isochore
chr9	39379250	39551456	172206	LOC653501, ZNF658B	Unknown
chr9	39829606	39961804	132198	FAM75A2, FAM75A1, FAM74A1	Unknown
chr9	41497718	41635419	137701	FAM75A5, FAM75A7, LOC653501, ZNF658B	Unknown
chr9	42743905	42847394	103489	LOC286297	Isochore_border
chr10	46799214	46907775	108561	FAM35B	Isochore
chr10	48185336	48300420	115084	LOC642826, AGAP9, FAM25B, FAM25G, FAM25C, ANXA8, ANXA8 L1	Isochore_border
chr16	33142890	33293778	150888	TP53TG3, TP53TG3C, TP53TG3B	Isochore_border
chrX	52098738	52395914	297176	XAGE2, XAGE2B, XAGE1B, XAGE1A, XAGE1D, XAGE1C, XAGE1E	Unknown
chrX	52445914	52568230	122316	XAGE1A, XAGE1C, XAGE1E, XAGE1D, XAGE1B	Isochore_border
chrY	4834281	4935713	101432	PCDH11Y	Isochore_border
chrY	5012892	5205540	192648	PCDH11Y	Unknown
chrY	5274434	5421065	146631	PCDH11Y	Isochore_border
chrY	6074690	6422524	347834	TTTY23, TTTY23B, TSPY2, TTTY1B, TTTY1, TTTY2B, TTTY2, TTTY21, TTTY21B, TTTY7B, TTTY7, TTTY8B,TTTY8	Isochore
chrY	9381846	9492957	111111	RBMY3AP	Isochore
chrY	9524503	9768115	243612	TTTY8, TTTY8B, TTTY7B, TTTY7, TTTY21, TTTY21B, TTTY2B, TTTY2, TTTY1, TTTY1B, TTTY22, TTTY23,TTTY23B	Isochore
chrY	14691127	14804076	112949	TTTY15	Isochore_border
chrY	19563894	20143885	579991	FAM41AY1, FAM41AY2, LINC00230B, LINC00230A, XKRY, XKRY2, CDY2B, CDY2A	Unknown
chrY	20193885	20834702	640817	XKRY, XKRY2, LINC00230A, LINC00230B, FAM41AY1, FAM41AY2, HSFY2, HSFY1,TTTY9B, TTTY9A	Unknown
chrY	20837553	21080706	243153	TTTY9B, TTTY9A, HSFY2, HSFY1, NCRNA00185	Unknown
chrY	22564778	22665261	100483	TTTY10	Unknown
chrY	23473201	23580342	107141	RBMY2EP	Isochore_border
chrY	23634362	23838234	203872	RBMY1B, RBMY1A1, RBMY1E, RBMY1D, TTTY13	Isochore_border
chrY	23993156	24359930	366774	RBMY1A1, RBMY1D, RBMY1B, RBMY1E, PRY, PRY2, TTTY6, TTTY6B, RBMY1F, RBMY1J	Isochore_border
chrY	24500602	24620459	119857	RBMY1F, RBMY1J, TTTY6B, TTTY6	Unknown
chrY	24620459	28160890	3540431	PRY, PRY2, TTTY17B, TTTY17C,TTTY17A, TTTY4C, TTTY4B, TTTY4, BPY2B, BPY2, BPY2C, DAZ1, DAZ4, DAZ3, DAZ2, TTTY3B, TTTY3, CDY1, CDY1B, CSPG4P1Y, GOLGA2P2Y, GOLGA2P3Y	Isochore_border
chr9	42027732	42145811	118079		Isochore_border
chr9	44466205	44651655	185450		Isochore_border
chr9	45128500	45250203	121703		Isochore_border
chr9	65632583	65745692	113109		Isochore_border
chrY	3016123	3134221	118098		Isochore
chrY	3179117	3359419	180302		Isochore_border
chrY	3833777	3966707	132930		Unknown
chrY	3966708	4346934	380226		Unknown
chrY	4466077	4593373	127296		Unknown
chrY	4593411	4807708	214297		Unknown
chrY	6482140	6677618	195478		Isochore_border
chrY	7401836	7548914	147078		Unknown
chrY	8214827	8334874	120047		Isochore_border
chrY	15039955	15234829	194874		Unknown
chrY	18248698	18381734	133036		Unknown
chrY	18390543	18560004	169461		Isochore_border
chrY	19375294	19500106	124812		Unknown
chrY	22214221	22369679	155458		Isochore_border
chrY	22419679	22564743	145064		Isochore_border
chrY	23241568	23361665	120097		Isochore_border
chrY	28160891	28509481	348590		Isochore_border

### Pathway and functional analyses

The genes identified in the CSFRs/CVFRs were used to analyze their enrichment of biological functions through the Database for Annotation, Visualization and Integrated Discovery (DAVID, http://david.abcc.ncifcrf.gov/tools.jsp).

### Isochore characterization

Isochore is a large region of DNA sequence which has a relatively uniform degree in its GC content [8]. We use 100 kb as the length of flank region and 2% GC difference as indicator to identify isochore, isochore border and unknown region among SNP free regions. All SNP free regions in this study are longer than 100 kb. CSFRs are identified as isochore if its GC content is 2% greater or lower than both right and left regions. CSFRs are identified as isochore border if the difference of GC content between two flank regions is greater than 2%, and GC-content difference between left flank and right flank region is greater than GC-content difference between CSFR and its flank regions. Unknown region means CSFR is neither isochore nor isochore border.

## Results

### CSFRs and CVFRs identification

We identified 50 CSFRs distributed across eight chromosomes: chr1, chr2, chr7, chr9, chr10, chr16, chrX, and chrY. The Y chromosome carried the majority of these regions–33 in total (Table 2). After excluding the CNV regions, we identified 20 CVFRs distributed across two chromosomes: chrX and chrY. The Y chromosome still carried the majority, with 18 regions (Table 3).

**Table 3:** List of 20 common variant-free regions containing 20 genes.

chr	CVFR_start	CVFR_end	CVFR_size	gene_name
chrX	52098738	52231295	132557	XAGE2, XAGE2B
chrX	52267361	52395914	128553	XAGE2, XAGE2B
chrY	4834281	4935713	101432	PCDH11Y
chrY	4935714	5205540	269826	PCDH11Y
chrY	5274434	5421065	146631	PCDH11Y
chrY	9524503	9640365	115862	TTTY8, TTTY8B, TTTY7B, TTTY7, TTTY21,
				TTTY21B, TTTY2B, TTTY2, TTTY1, TTTY1B
				TTTY22
chrY	20228333	20599266	370933	XKRY, XKRY2, LINC00230A, LINC00230B
				FAM41AY1, FAM41AY2
chrY	3016123	3134221	118098	
chrY	3179117	3359419	180302	
chrY	4114366	4346934	232568	
chrY	4466077	4593373	127296	
chrY	4593411	4807708	214297	
chrY	6577215	6677618	100403	
chrY	8214827	8334874	120047	
chrY	15039955	15234829	194874	
chrY	17559652	17661377	101725	
chrY	18248698	18381734	133036	
chrY	18390543	18560004	169461	
chrY	19375294	19500106	124812	
chrY	23247004	23361665	114661	

We checked our results in the Affymetrix SNP Array 6.0 by its annotation data. Among the CSFRs, we found 25 SNPs' information in the annotation file, and only four of them had non-zero minor allele frequency: rs11681529, rs2571764, rs2874557, and rs35516764. The other 20 are monomorphic for HapMap four populations (Caucasian, African, Chinese and Japanese). Therefore, we concluded that most of these 50 large genomic regions has not been covered properly by the Affymetrix 6.0 Array at least in those major populations investigated.

### Genes in CSFRs and CVFRs and their functional enrichment

Ninety-seven genes overlapped with 28 of the 50 CSFRs (56%) (Table 2). DAVID was used to test whether the annotations of this set of genes were over presented with particular GO terms [9]. They were highly enriched with biological pathways involved with sexual reproduction, spermatogenesis, male gamete generation, gamete generation, multicellular organism reproduction, and reproductive processes in a multicellular organism (p<0.05 and FDR q<0.05, Table 4). The gene set included a number of gene previously reported to be related to reproduction, including *DAZ1* [10], [11], *BPY2* [12], *TSPY2* [11], *CDY1* [13], *CDY2A* [13] and *RBMY1* [11]. A gr/gr deletion polymorphism on Y chromosome of those CSFRs has also been suggested to be a risk factor of spermatogenic impairment in some populations [14], [15].

**Table 4:** Top 6 GO terms from the functional annotation analysis of 97 CSFR genes by DAVID.

Category	Term	Count	%	P-Value	FDR
GOTERM_BP_FAT	sexual reproduction^1^	9	14.8	0.00000003	0.000033
GOTERM_BP_FAT	Spermatogenesis^2^	8	13.1	0.000000047	0.000052
GOTERM_BP_FAT	male gamete generation^2^	8	13.1	0.000000047	0.000052
GOTERM_BP_FAT	gamete generation^2^	8	13.1	0.00000026	0.00028
GOTERM_BP_FAT	multicellular organism reproduction^2^	8	13.1	0.0000011	0.0012
GOTERM_BP_FAT	reproductive process in a multicellular organism^2^	8	13.1	0.0000011	0.0012

1 gene included RBMY1A1, RBMY1B, RBMY1J, RBMY1F, XKRY, XKRY2, BPY2C, BPY2B, BPY2, CDY1, CDY1B, CDY2B, CDY2A, DAZ2, DAZ3, DAZ4, DAZ1, and TSPY2.

2 gene included RBMY1A1, RBMY1B, RBMY1J, RBMY1F, BPY2C, BPY2B, BPY2, CDY1, CDY1B, CDY2B, CDY2A, DAZ2, DAZ3, DAZ4, DAZ1, and TSPY2.

Twenty genes were overlapped with seven of the 20 CVFRs (35%) (Table 3). DAVID was also performed on these 20 genes. However, these genes were not enriched in any biological functions.

### SNP-free regions from personal genome sequencing and segmental duplications

We further explored those SNP-free regions in personal genome variant data. Rare variants were detected in most of the CSFRs or CVFRs. Only one region on X chromosome (chrX: 52,267,361-52,395,914) left. We also examined this region in updated dbSNP database (dbSNP137, http://www.ncbi.nlm.nih.gov/). Two more common SNPs were deteceted (rs201652812 and rs199865557). After subtract them, the left region was 105 kb (chrX: 52,290,698-52,395,914), which was the finally region not containing any known variant in all of the genome-wide sequencing data that we were able to collect. *XAGE2* and its splicing isoforms were harbored in this region.

We next tested this final region in segmental duplication database from Eichler's lab (http://eichlerlab.gs.washington.edu/database.html) [7], and found it was overlapped with one of the segmental duplication regions.

We found that 49 CSFRs did carry SNPs in the Genomes Unzipped and Personal Genome Variation tracks. And the left X chromosome region did not contain any SNPs but overlapped with segmental duplication region.

### Twenty-four CSFRs are isochore borders

To dig out the sequence properties of 50 CSFRs, we characterized those regions by GC content. Different GC contents can separated DNA sequences into compositionally fairly homogeneous regions [8]. By comparing GC contents between CSFRs and their flanking regions, we found that twenty-four CSFRs belong to isochore border regions, seven belong to isochore regions, and eighteen are unknown regions (Table 2, Table S1).

## Discussion

We performed a thorough search for large genomic regions that are free of common variants in dbSNP and we found 50 CSFRs and 20 CVFRs. Most of these variations free regions located on Y chromosome. Genes in the CSFRs were highly enriched for activities related to reproduction. Further investigation in the sequencing of personal genomes found most of the CSFRs (49 out of 50) did contain rare SNPs, suggesting those regions have not been covered well in the existing common variants sequencing projects, like the 1000 Genomes Project.

GWAS is one the most infusive common variants sequencing projects, but important finding might be missed because of its poor coverage of rare variants. Recently, two fertility GWAS studies were conducted but failed to find SNPs on sex chromosomes [16], [17]. Both studies used Affymetrix GWAS platforms that we evaluated in this study. However, both sex chromosomes have long been implicated in infertility, specifically in spermatogenic damage in mouse models and in human candidate gene/region studies [18]. Our study found that those genomic regions free of common variants regions carrying many genes important to reproduction. With those important candidate genes missing, we must be cautious of analyzing fertility-related GWASs, which may produce false negatives.

The most reliable CVFR call contains the *XAGE2* and its isoforms, which belong to *XAGE* subfamily. *XAGE2* is strongly expressed in normal testes, and in some tumor [19]. Because genotyping platforms cannot fully cover structural variations such as segmental duplication, we further applied structural variations filtering analysis, and observed *XAGE* region was overlapped with segmental duplication. Based on these observations, we concluded that the observation of variant free regions is more a coverage problem with the current versions of dbSNP and existing GWAS assay platforms than a lack of assayable variation. When more genomes are sequenced, we may end up with proper coverage of complete human genome by common SNPs.

We mapped our SNPs on dbSNP build 135 and regions on GRCh37.p10 (hg19) assembly reference, which is the most accurate alignment version and with all current genome knowledge available. Comparing to old versions, hg19 changed many genomic coordinates and included alternate haplotype assemblies for chr6 (7 haplotypes), chr4 (1 haplotype), and chr17 (1 haplotype). Different versions can be converted by liftOver software (http://genome.ucsc.edu/cgi-bin/hgLiftOver). More details of differences in each version are provided in NCBI (http://www.ncbi.nlm.nih.gov/genome/guide/human/release_notes.html).

Further study can focus on the sequence properties of those regions, and their conservative across species. Isochores are spatially heterogeneous in mammalian genome and varies in replication timing, gene richness, recombination rate, etc [20], [21], [22]. Natural selection is the most plausible explanation for formation and maintenance of isochores [20]. We observed nearly half of CSFRs are isochores and isochore border regions, which is a hint that these CSFRs may be under different selection pressure from its neighboring regions. To further test selection pressure, we mapped those regions to chimpanzee and mouse by Synteny analysis from Ensembl (http://useast.ensembl.org/Homo_sapiens/Location/Synteny?r=6:133017695-133161157), and found only 6 genes (*RGPD5*, *RGPD6*, *GATSL2*, *FAM25G*, *HSFY1*, *HSFY2*) can map to unique regions in the other two species. Next we applied dN/dS ratio test, the ratio of substitution rates at non-synonymous and synonymous sites, and found that human genes under more purify selection than chimpanzee genes (paired T test, p = 0.01, Table S2). Those results suggest that natural selection seems to be the major evolutionary force behind these variant-free regions.

In summary, by searching large genomic regions free of common variants for the first time, we identified tens of common variations free regions, and most of them were located on the X and Y chromosomes. The genes located in CSFRs are enriched for fertility. Incorporating personal genome data, only one region was still free of variants and harbored gene *XAGE2*, indicating most of the detections due to low coverage of rare variations. Future deep sequencing from more individuals and redesigning GWAS arrays should improve our understanding of the variability of these regions and their functional importance.

## Supporting Information

Table S1 **Isochore characterization of 50 CSFRs.**
(DOC)

Table S2 **Evolution pressure of conserved genes by dN/dS ratio test.**
(DOC)
