# Supplementary material for: Large Genomic Region Free of GWAS-Based Common Variants Contains Fertility-Related Genes
Source: PLoS One. 2013 Apr 17;8(4):e61917. doi: 10.1371/journal.pone.0061917 (PMC3629113; doi:10.1371/journal.pone.0061917)
Supplement: Table S1 — Isochore characterization of 50 CSFRs. (DOC) [file pone.0061917.s001.doc]

**Table S1. Isochore characterization** of 50 CSFRs

| **CHR** | **start** | **end** | **GC%** | **GC _left^1^** | **GC _right^2^** | **Avg_GC**  **Region&&**  **Flanks^3^** | **Diff_GC**  **Left&&**  **Right^4^** | **Region_type** |
| --- | --- | --- | --- | --- | --- | --- | --- | --- |
| chr10 | 46799214 | 46907775 | 0.377 | 0.428 | 0.463 | 0.069 | 0.035 | isochore |
| chr2 | 110524226 | 1.11E+08 | 0.408 | 0.446 | 0.476 | 0.053 | 0.03 | isochore |
| chr7 | 74765724 | 74866460 | 0.519 | 0.47 | 0.474 | 0.047 | 0.004 | isochore |
| chrY | 6074690 | 6422524 | 0.413 | 0.37 | 0.358 | 0.049 | 0.012 | isochore |
| chrY | 9381846 | 9492957 | 0.394 | 0.456 | 0.437 | 0.052 | 0.019 | isochore |
| chrY | 9524503 | 9768115 | 0.43 | 0.385 | 0.376 | 0.049 | 0.009 | isochore |
| chrY | 3016123 | 3134221 | 0.375 | 0.345 | 0.355 | 0.024 | 0.01 | isochore |
| chr10 | 48185336 | 48300420 | 0.468 | 0.396 | 0.476 | 0.04 | 0.08 | isochore_border |
| chr16 | 33142890 | 33293778 | 0.419 | 0.39 | 0.46 | 0.035 | 0.07 | isochore_border |
| chr1 | 145883118 | 1.46E+08 | 0.419 | 0.39 | 0.444 | 0.027 | 0.054 | isochore_border |
| chr2 | 111191098 | 1.11E+08 | 0.408 | 0.467 | 0.403 | 0.032 | 0.064 | isochore_border |
| chr9 | 42743905 | 42847394 | 0.405 | 0.37 | 0.399 | 0.021 | 0.029 | isochore_border |
| chr9 | 42027732 | 42145811 | 0.345 | 0.392 | 0.338 | 0.027 | 0.054 | isochore_border |
| chr9 | 44466205 | 44651655 | 0.401 | 0.398 | 0.432 | 0.017 | 0.034 | isochore_border |
| chr9 | 45128500 | 45250203 | 0.4 | 0.465 | N.A. | N.A. | N.A. | N.A. |
| chr9 | 65632583 | 65745692 | 0.387 | 0.388 | 0.42 | 0.017 | 0.032 | isochore_border |
| chrX | 52445914 | 52568230 | 0.428 | 0.395 | 0.434 | 0.019 | 0.039 | isochore_border |
| chrY | 4834281 | 4935713 | 0.359 | 0.371 | 0.35 | 0.011 | 0.021 | isochore_border |
| chrY | 5274434 | 5421065 | 0.347 | 0.373 | 0.351 | 0.015 | 0.022 | isochore_border |
| chrY | 14691127 | 14804076 | 0.397 | 0.429 | 0.368 | 0.03 | 0.061 | isochore_border |
| chrY | 23473201 | 23580342 | 0.379 | 0.374 | 0.399 | 0.013 | 0.025 | isochore_border |
| chrY | 23634362 | 23838234 | 0.407 | 0.401 | 0.378 | 0.017 | 0.023 | isochore_border |
| chrY | 23993156 | 24359930 | 0.408 | 0.432 | 0.385 | 0.023 | 0.047 | isochore_border |
| chrY | 24620459 | 28160890 | 0.394 | 0.423 | 0.386 | 0.018 | 0.037 | isochore_border |
| chrY | 3179117 | 3359419 | 0.356 | 0.373 | 0.352 | 0.011 | 0.021 | isochore_border |
| chrY | 6482140 | 6677618 | 0.357 | 0.357 | 0.396 | 0.02 | 0.039 | isochore_border |
| chrY | 8214827 | 8334874 | 0.394 | 0.398 | 0.427 | 0.018 | 0.029 | isochore_border |
| chrY | 18390543 | 18560004 | 0.378 | 0.385 | 0.357 | 0.014 | 0.028 | isochore_border |
| chrY | 22214221 | 22369679 | 0.429 | 0.372 | 0.431 | 0.029 | 0.059 | isochore_border |
| chrY | 22419679 | 22564743 | 0.402 | 0.432 | 0.373 | 0.029 | 0.059 | isochore_border |
| chrY | 23241568 | 23361665 | 0.39 | 0.418 | 0.369 | 0.024 | 0.049 | isochore_border |
| chrY | 28160891 | 28509481 | 0.386 | 0.368 | 0.39 | 0.011 | 0.022 | isochore_border |
| chr9 | 39379250 | 39551456 | 0.396 | 0.405 | 0.41 | 0.011 | 0.005 | unknown |
| chr9 | 39829606 | 39961804 | 0.403 | 0.386 | 0.381 | 0.02 | 0.005 | unknown |
| chr9 | 41497718 | 41635419 | 0.404 | 0.388 | 0.404 | 0.008 | 0.016 | unknown |
| chrX | 52098738 | 52395914 | 0.413 | 0.406 | 0.406 | 0.007 | 0 | unknown |
| chrY | 5012892 | 5205540 | 0.365 | 0.35 | 0.366 | 0.008 | 0.016 | unknown |
| chrY | 19563894 | 20143885 | 0.395 | 0.376 | 0.381 | 0.017 | 0.005 | unknown |
| chrY | 20193885 | 20834702 | 0.391 | 0.383 | 0.392 | 0.005 | 0.009 | unknown |
| chrY | 20837553 | 21080706 | 0.376 | 0.385 | 0.399 | 0.016 | 0.014 | unknown |
| chrY | 22564778 | 22665261 | 0.373 | 0.388 | 0.383 | 0.013 | 0.005 | unknown |
| chrY | 24500602 | 24620459 | 0.414 | 0.39 | 0.4 | 0.019 | 0.01 | unknown |
| chrY | 3833777 | 3966707 | 0.352 | 0.351 | 0.348 | 0.003 | 0.003 | unknown |
| chrY | 3966708 | 4346934 | 0.358 | 0.352 | 0.368 | 0.008 | 0.016 | unknown |
| chrY | 4466077 | 4593373 | 0.354 | 0.374 | 0.362 | 0.014 | 0.012 | unknown |
| chrY | 4593411 | 4807708 | 0.364 | 0.355 | 0.367 | 0.006 | 0.012 | unknown |
| chrY | 7401836 | 7548914 | 0.397 | 0.404 | 0.418 | 0.014 | 0.014 | unknown |
| chrY | 15039955 | 15234829 | 0.386 | 0.385 | 0.378 | 0.005 | 0.007 | unknown |
| chrY | 18248698 | 18381734 | 0.384 | 0.382 | 0.377 | 0.005 | 0.005 | unknown |
| chrY | 19375294 | 19500106 | 0.374 | 0.385 | 0.381 | 0.009 | 0.004 | unknown |

^1^Percentage of GC content in the left flank region of CSFR; ^2^Percentage of GC content in the right flank region of CSFR; ^3^GC-content difference between SNP-free region and the average of its both side flanking regions; ^4^GC-content difference between left flanking and right flanking region.
