# Supplementary material for: Large Genomic Region Free of GWAS-Based Common Variants Contains Fertility-Related Genes
Source: PLoS One. 2013 Apr 17;8(4):e61917. doi: 10.1371/journal.pone.0061917 (PMC3629113; doi:10.1371/journal.pone.0061917)
Supplement: Table S2 — Evolution pressure of conserved genes by dN/dS ratio test. (DOC) [file pone.0061917.s002.doc]

**Table S2**. Evolution pressure of conserved genes by dN/dS ratio test

| Mouse_gene | Human_gene | Chimpanzee_gene | Human_to_mouse(dn/ds) | Chimpanzee_to_mouse(dn/ds) |
| --- | --- | --- | --- | --- |
| ENSMUSG00000003226 | ENSG00000015568 | ENSPTRG00000012330 | 0.28 | 0.30 |
| ENSMUSG00000003226 | ENSG00000183054 | ENSPTRG00000012330 | 0.28 | 0.30 |
| ENSMUSG00000015944 | ENSG00000198750 | ENSPTRG00000019301 | 0.03 | 0.03 |
| ENSMUSG00000043681 | ENSG00000197910 | ENSPTRG00000029846 | 0.26 | 0.28 |
| ENSMUSG00000045336 | ENSG00000172468 | ENSPTRG00000028817 | 0.94 | 0.99 |
| ENSMUSG00000045336 | ENSG00000169953 | ENSPTRG00000028817 | 0.94 | 0.99 |
